# Identification of a large *DPYD* gene deletion undetected by standard genotyping in a patient with severe capecitabine-related toxicity: a case report

**DOI:** 10.3389/fphar.2026.1866621

**Published:** 2026-08-04

**Authors:** Marco Donatello Delcuratolo, Maria Grazia Rodriquenz, Franco Morelli, Tiziana Pia Latiano, Lucia Anna Muscarella, Simona Caporilli, Lorenza Pasqualini, Elisabetta De Santis, Vincenzo Giambra, Antonio Rinaldi, Grazia Ciavarella, Matteo Floris, Roberta Murru, Alessia Mascia, Elena De Mattia, Giovanni Canil, Giuseppe Fania, Ana Pop, Massimo Carella, André van Kuilenburg, Erika Cecchin, Giuseppe Miscio

**Affiliations:** 1 Oncology Unit, Fondazione IRCCS Casa Sollievo della Sofferenza, San Giovanni Rotondo (FG), Italy; 2 Lung Cancer Research Unit, Fondazione IRCCS Casa Sollievo della Sofferenza, San Giovanni Rotondo (FG), Italy; 3 Life Sciences and Diagnostic Group, Agilent Technologies Italia S.p.A. Cernusco sul Naviglio, Milan, Italy; 4 Hematopathology Unit, Institute for Stem Cell Biology, Regenerative Medicine and Innovative Therapeutics (ISBReMIT), Fondazione IRCCS Casa Sollievo della Sofferenza, San Giovanni Rotondo (FG), Italy; 5 Medicina Trasfusionale e Laboratorio Analisi, Fondazione IRCCS Casa Sollievo Della Sofferenza, San Giovanni Rotondo, Italy; 6 Department of Medical Sciences and Public Health, University of Cagliari, Cagliari, Italy; 7 Department of Medical Genetics, Binaghi Hospital, ASL Cagliari, Cagliari, Italy; 8 Unit of Oncology and Molecular Pathology, Department of Biomedical Sciences, University of Cagliari, Cagliari, Italy; 9 Experimental and Clinical Pharmacology, Centro di Riferimento Oncologico di Aviano (CRO), IRCCS, Aviano (PN), Italy; 10 Amsterdam UMC, Vrije Universiteit Amsterdam, Laboratory Genetic Metabolic Diseases, Amsterdam, Netherlands; 11 Medical Genetics Unit, Fondazione IRCCS Casa Sollievo della Sofferenza, San Giovanni Rotondo (FG), Italy; 12 Cancer Center Amsterdam, Amsterdam, Netherlands

**Keywords:** case report, DPD activity, *DPYD* gene, fluoropyrimidine toxicity, next-generation sequencing

## Abstract

**Background:**

Fluoropyrimidines are widely used in the treatment of multiple neoplasms, but their use is limited by the risk of severe toxicity in patients with dihydropyrimidine dehydrogenase (DPD) deficiency. Current clinical guidelines recommend *DPYD* genotyping as pre-treatment screening for a limited number of common variants; however, this approach fails to identify all patients at risk.

**Case Description:**

We describe the case of a man with resected gastric adenocarcinoma, wild type for the *DPYD* recommended variants, who developed early-onset, life-threatening toxicity shortly after few days of adjuvant capecitabine-based chemotherapy at full dose. The clinical course was characterized by grade 4 gastrointestinal toxicity complicated by septic shock, which required prolonged hospitalization and led to the definitive discontinuation of chemotherapy. Extensive Next-Generation Sequencing (NGS) revealed a heterozygous deletion spanning from exons 5 to 20 (for a total of about 500 Kb) of the *DPYD* gene. The presence of this deletion was validated using Single Nucleotide Polymorphism (SNP) Array. Functional analysis, based on assessment of DPD activity in peripheral blood mononuclear cells (PBMCs) and plasma concentrations of uracil and dihydrouracil, confirmed the impaired DPD function.

**Conclusion:**

This case highlights the limitations of routine *DPYD* genotyping strategies and emphasizes the clinical relevance of rare structural variants as an underestimated cause of severe fluoropyrimidine-related toxicity. The use of comprehensive NGS approach in pharmacogenetic diagnostics should be considered as a step forward in the identification of patients at risk of fluoropyrimidines-related toxicity particularly in the context of adjuvant therapy, where it is essential to strike a balance between therapeutic benefits and unacceptable toxicity.

## 1 Introduction

Fluoropyrimidines, including 5-fluorouracil (5-FU) and the oral prodrug capecitabine, are widely used both as single agents and as part of combination regimens in various solid tumors, such as gastrointestinal, breast and head/neck cancers (Longley et al., 2003).

Dihydropyrimidine dehydrogenase (DPD), encoded by the *DPYD* gene, is a key enzyme involved in the metabolism of 5-FU and its prodrugs, converting more than 80% of 5-FU into inactive metabolites (Diasio and Harris, 1989). DPD deficiency, which affects about the 3%–7% of Caucasian population, leads to excessive concentrations of the active metabolites of 5-FU and consequent severe toxicity (myelosuppression, diarrhea, mucositis, neurotoxicity), which is fatal in approximately 1% of patients (Knikman et al., 2021).

Various genotyping or phenotyping approaches have been developed to assess DPD deficiency and are routinely used in clinical practice to individualize dosing of fluoropyrimidines and reduce the incidence of severe toxicity (Henricks et al., 2018a; Bignucolo et al., 2023), particularly grade 4–5 events, which DPYD pathogenic variants strongly predict (Le Teuff et al., 2024).

The European Medicines Agency (EMA) recommends two methods for DPD deficiency testing before starting cancer treatment including fluoropyrimidine: phenotyping by measuring pre-treatment plasmatic uracil concentration or genotyping for the presence of *DPYD* risk variant alleles, namely c.1905 + 1G>A (rs3918290), c.1679T>G (rs55886062), c.2846A>T (rs67376798), c.1129-5923C>G (rs75017182) (European Medicines Agency, 2020). Although phenotyping appears to be a more comprehensive approach to identify patients with impaired enzymatic activity as compared to genotyping (Etienne-Grimaldi et al., 2023), it is currently challenged by several pre-analytical issues, which make a wide routine application difficult (Knikman et al., 2025). On the other hand, genotyping of a limited panel, as proposed by current pharmacogenetic guidelines, limits the efficiency in identifying all the patients at risk of severe toxicity. Population-specific or rare *DPYD* variants, which are not always detected by standard genotyping testing, can lead to severe and potentially life-threatening toxicity from fluoropyrimidines (De Mattia et al., 2022; De Mattia et al., 2025; De Mattia et al., 2024). New sequencing technologies, such as NGS, may contribute to capture rare genetic alterations (Larrue et al., 2024).

In this report, we present the case of a patient with gastric adenocarcinoma, wild-type for the 4 most common *DPYD* variants, treated with full-dose capecitabine-based chemotherapy in the adjuvant setting, who experienced severe fluoropyrimidine-related toxicity due to a large heterozygous deletion in *DPYD*.

## 2 Clinical presentation

A 67-year-old Caucasian man with a history of ascending aortic dilation, type 2 diabetes mellitus and benign prostatic hypertrophy, with no significant family history of cancer, was admitted to the Casa Sollievo della Sofferenza Hospital in San Giovanni Rotondo (Italy) in August 2024, reporting 2 months of early satiety and an unintentional weight loss of 5 kg in 3 months. Following a gastroscopy and a contrast-enhanced CT scan of the chest and abdomen, the patient was diagnosed with clinical stage IB diffuse-type gastric cancer (cT2N0). Therefore, patient was offered neoadjuvant chemotherapy with FLOT regimen (*5-fluorouracil*, *oxaliplatin*, *docetaxel*), which he refused. In September 2024, the patient underwent a laparoscopic subtotal gastrectomy with D2 lymphadenectomy and Billroth II reconstruction. Histopathological examination revealed a diffuse-type gastric carcinoma with signet-ring cell features, staged as pT3N3a, with unaffected resection margins. After surgery, a post-operative CT scan showed no local recurrence or distant metastases but considering the high risk of recurrence, adjuvant therapy with CAPOX (*oxaliplatin* 130 mg/mq day 1, *capecitabine* 1,000 mg/mq for 14 days every 3 weeks) was planned for a total of 8 cycles. According to the Italian pharmacogenetic guidelines (AIOM-SIF Working Group, 2024), a targeted pharmacogenetic analysis by *DPYD* genotyping (for the four most common variants: c.1905 + 1G>A (DPYD*2A, rs3918290), c.1679T>G (DPYD*13, rs55886062), c.2846A>T (rs67376798), and c.1129-5923C>G (rs75017182) was performed without revealing any pathogenic variants. On 18 November 2024, chemotherapy was started as planned.

After 8 days of capecitabine treatment the patient was admitted to the emergency department for severe and persistent diarrhea (12–15 stools per day) (G4 according to CTCAE, version 5.0) unresponsive to antidiarrheal therapy, nausea G3 and vomiting G2, accompanied by significant clinical deterioration. Blood tests showed hypokalemia G2 and hypoalbuminemia G2, without any significant alteration in blood count. The patient was then admitted to the Oncology Department to continue the appropriate treatment and *capecitabine* was discontinued. *Salmonella* and *Shigella* infection, as well as *Clostridium difficile* toxin genes were excluded. Gastroscopy as well as chest and abdominal CT scans did not highlight any sign of early postoperative recurrence and tumour markers were normal. A significant reduction in ejection fraction – compared to the previous examination (45%) – was detected. The cardiological evaluation thus attributed the reduction in ejection fraction to the patient’s severe hypovolemia. Hydration and intravenous potassium supplementation were initiated, along with parenteral nutrition. *DPYD* genotyping was repeated, confirming the absence of any pathogenic variants, including *DPYD* c.2194G>A (rs1801160) recommended by the Italian guidelines in the post-toxicity setting (AIOM-SIF Working Group, 2024).

On the tenth day of hospitalization, despite supportive therapy and gradual improvement in diarrhea, nausea and vomiting to grade G1, he developed significant hypotension without fever, reduced urine output, dyspnea (91% saturation in room air) and tachypnoea. Arterial blood gas analysis showed a pO2 of 75 mmHg, normal pCO2 and pH, and an increase in lactate to 3.5 mmol/L. Laboratory tests revealed elevated levels of CRP (27,9 mg/L) and procalcitonin (174,8 ng/mL), with leukocytosis (18 × 10^9^/L with neutrophils 16.8 × 10^9^/L) and an increase in creatinine to 1.9 mg/dL. Blood cultures were taken from the central vascular access and peripheral blood, both of which tested positive for *Klebsiella pneumoniae*. In light of the septic shock, following the recommendation of the resuscitation team, norepinephrine 0.06 mcg/kg/min was administered. Antibiotic therapy was initially set empirically with piperacillin/tazobactam and, subsequently, with the persistence of elevated inflammation indices and the results of the antibiogram, it was switched to meropenem/vaborbactam and linezolid. In addition, severe thrombocytopenia associated with the infection necessitated the transfusion of three units of platelets.

During the subsequent days of hospitalization, hemodynamics and inflammatory markers gradually improved, allowing de-escalation to ceftazidime monotherapy and tapering of norepinephrine; in addition, episodes of diarrhea were further decreasing.

After 25 days of hospitalization, the patient was discharged in significantly improved general condition, afebril, without diarrhea and with negative inflammation indices. The patient had regained oral feeding without reporting nausea or vomiting and with a recovery in platelet and potassium values. Due to the severe toxicity reported, adjuvant chemotherapy was definitively stopped and a strict follow-up was planned, including clinical evaluation, blood tests and CT-scan every 3–4 months for the first year. From a psychological point of view, there has been a marked improvement in mood, with increased optimism and satisfaction after returning to daily routines.

Additional pharmacogenetic analyses were subsequently requested, and a targeted NGS approach focusing on the *DPYD* gene was performed to either confirm a gene-specific involvement or rule out the possibility that the adverse drug reaction was genotype-related.

## 3 Results

### 3.1 Real-time polymerase chain reaction (RTq-PCR)

Pre-emptive real-time PCR (RT-PCR) screening of the *DPYD* gene was conducted in accordance with the AIOM-SIF 2019 (and 2024 update) guidelines (AIOM-SIF Working Group, 2024). The analyzed variants included *DPYD* c.1905 + 1G>A, c.1679T>G, c.2846A>T, c.1129-5923 C>G, all showing a wild-type genotype, indicating the absence of pathogenic variants or clinically relevant variants. After the reported adverse drug reaction (ADR), the workflow was repeated with a new whole blood sample and including c.2194G>A variant (DPYD*6, rs1801160), as per SIF-AIOM guidelines, but RT-PCR genotyping confirmed the absence of the five tested variants.

Whole *DPYD* gene sequencing using a custom NGS pharmacogenetics panel was subsequently performed.

### 3.2 Next-generation sequencing (NGS)

**FIGURE 1:**
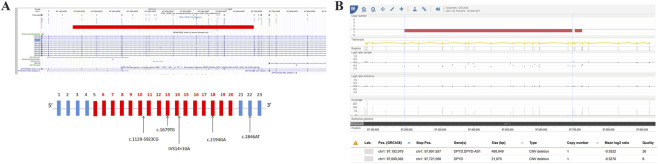
*DPYD* gene deletion. *DPYD* gene location of the 500 Kb deletion (NCBI RefSeq transcript NM_000110.4; ENSEMBL ENST00000370192.8 canonical isoform). Positions of the variants screened according to Italian AIOM-SIF recommendations (AIOM-SIF Working Group, 2024) are showed (**(A)**, lower panel). In red the extension of the deletion. **(B)** Visualization of the DPYD locus (GRCh38) showing copy number analysis based on normalized read depth. The CNV track (red) indicates an heterozygous deletion spanning a large genomic region. The log2 ratio plot for the sample shows a consistent shift toward negative values across the affected exons (mean log2 ratio ∼ −0.5), while the reference samples remain centered around zero, supporting a copy number loss.

The NGS analysis conducted using our pharmacogenetics panel revealed indeed the presence of a large heterozygous deletion in *DPYD* spanning about 500 kb, which includes the central part of the gene from exon 5 to exon 20 (Figure 1). The CNV ratios derived from the Alissa Reporter analysis showed a consistent reduction in normalized read depth across the affected region, with mean log2 ratio values of approximately −0.5, which is characteristic of a heterozygous deletion. In Figure 1B the log2 ratio plot of the sample shows a systematic shift toward negative values across the deleted interval, while reference samples remain centered around zero. The accompanying table reports genomic coordinates, gene annotation, and average log2 ratio values, all consistent with copy number loss. To further strengthen the interpretation, exon-level quantification obtained from the independent SeqOne analysis has also been included. The corresponding table (Supplementary Table S1) reports copy ratio values for each exon, showing a reduction ranging approximately from 0.43 to 0.60 across the affected region, in line with the expected ∼0.5 value for a heterozygous deletion. In contrast, flanking exons display ratios close to 1.0, supporting a normal diploid state. The consistency of these values across multiple consecutive exons supports the presence of a true biological event. It is noteworthy that the location of this deletion spans the gene segment that includes 4 of the 5 variants generally analyzed for *DPYD* gene screening; only the c.2846A>T variant, located in exon 22, is not included in the deleted region (Figure 1A). Overall, the deletion was identified using a validated read-depth–based approach; this finding was independently confirmed using a second bioinformatics platform (SeqOne); both analytical approaches rely on different implementations of coverage-based CNV detection, increasing confidence in the result obtained.

### 3.3 Single nucleotide polymorphism (SNP) array

To confirm the presence of the detected deletion in the patient, and to exclude the possibility of an NGS artifact, we collected a new whole blood sample and performed a fresh DNA extraction. This DNA was then analyzed using an alternative molecular technique specifically, a single nucleotide polymorphism (SNP) Array which offers a complementary and highly accurate method for the detection of large genomic deletions.

**FIGURE 2:**
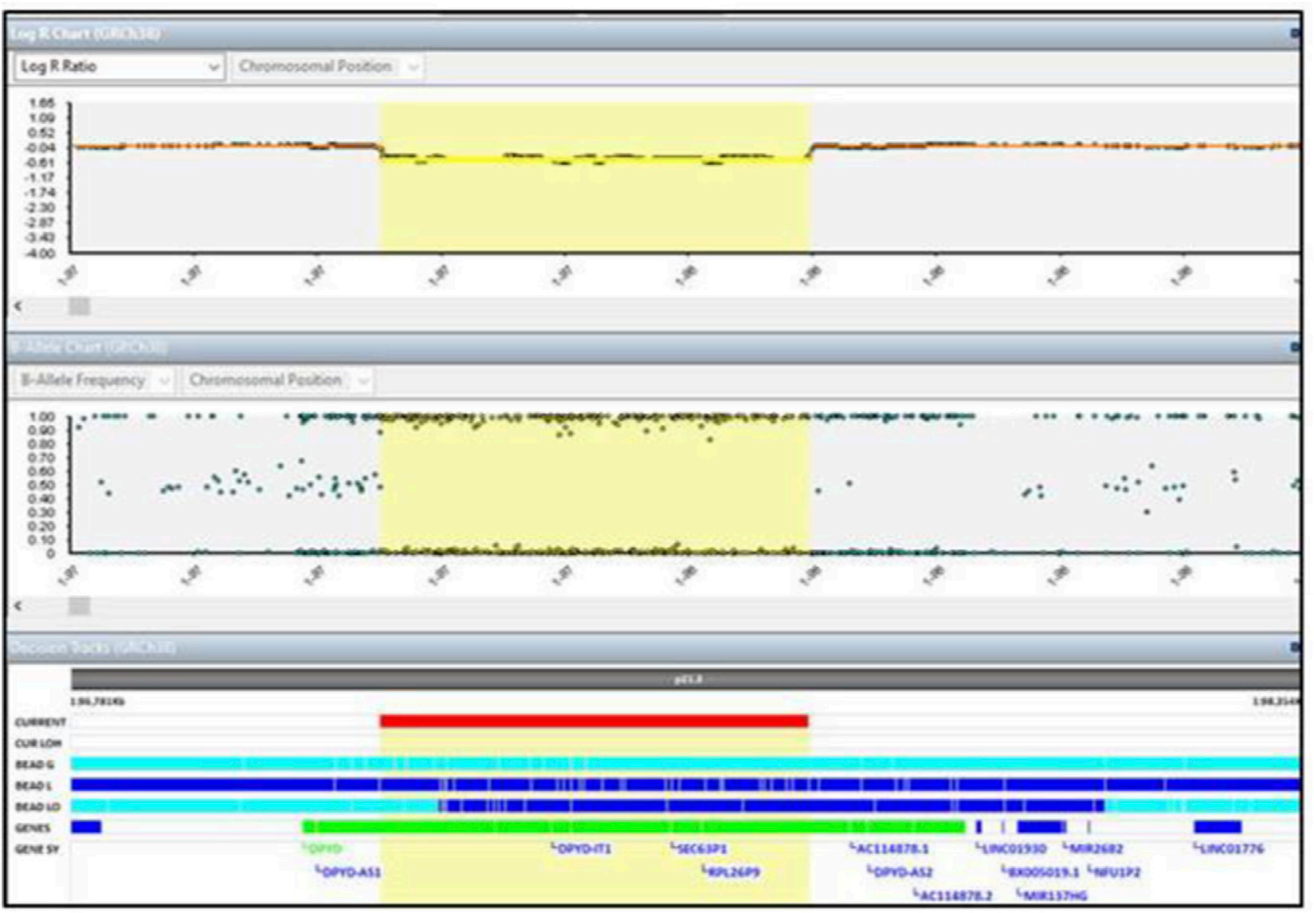
SNP-array analysis. The figure shows a schematic representation of the deletion in 1p21.3, from 97,176,283bp to 97,721,542bp encompassing the *DPYD* gene. The graph at the upper part of the figure represents the Log R value of the fluorescence intensity: it decreases to negative values in the deleted region. The graph in the middle represents the B Allele Frequency (BAF): it shows the presence of a single allele (A or B) within the deleted region. The graph at the lower part represents the deletion (red bar) compared to the entire gene (green bar).

The SNP array detected a 545.3 Kb deletion at chromosome 1p21.3 from the distal SNP at base pair 97176283 to the proximal SNP at base pair 97721542 [GRCh38] (Figure 2). The deletion includes 494 clones with an average Log R value of −0.481.This region encompasses *DPYD* gene associated with _Dihydropyrimidine dehydrogenase deficiency _(#274270_; _AR_) _and_ 5-fluorouracil toxicity _(#274270; AR). The entire gene *DPYD* is covered by 770 SNP clones and the deletion is completely within the gene, from exon 5 to exon 20.

### 3.4 DPD activity in peripheral blood mononuclear cells (PBMCs)

The DPD activity in peripheral blood mononuclear cells (PBMCs) of the patient was decreased compared to two healthy controls. As a matter of fact, DPD activity was reduced (49%) compared with that observed in healthy volunteers and was comparable to that reported in obligate heterozygotes (van Kuilen et al., 2002).

### 3.5 Dihydrouracil/uracil (DHU/U) ratio

The plasma concentrations of uracil (U) and dihydrouracil (DHU) measured in the patient and four control subjects (healthy individuals with a *DPYD* variant negative DNA) plasma samples are reported in Table 1. Patient samples were analyzed in duplicate and compared to controls. Based on published evidence, DPD deficiency was defined as plasma uracil concentrations >16 ng/mL and/or a DHU/U ratio <6 (Knikman et al., 2021; Contu et al., 2025; Capitain et al., 2020). Patient samples showed markedly increased plasma uracil concentrations (mean = 27.5 ng/mL) relative to dihydrouracil levels, together with a reduced DHU/U ratio (mean = 2.5), supporting impaired DPD activity (Table 1).

**TABLE 1:** Uracil and dihydrouracil plasma concentrations (ng/mL) and their DHU/U ratio.

Samples	Uracil (ng/mL)		Dihydrouracil (ng/mL)		DHU/U	
	Mean	St. dev	Mean	St. dev	Mean	St. dev
Patient (n = 1)	27.5*	0.7	68.3*	2.7	2.5	0.03
Controls (n = 4)	9.2	3.3	95.4	20.1	10.8	2.2

Plasma uracil and dihydrouracil concentrations were measured in the patient and control subjects.

The patient showed a marked increase in uracil relative to dihydrouracil, resulting in a reduced.

DHU/U ratio; this finding is suggestive of reduced DPD activity.

* Mean of duplicate sample.

## 4 Discussion

In this case report a case of severe capecitabine-related toxicity in a patient with a large *DPYD* deletion not detected by routine pre-treatment panel genotyping was described. This case highlights the limitations of current pharmacogenetic screening for the initiation of a fluoropyrimidine-based therapy and emphasizes the importance of integrating genomic and functional strategies in selected cases. Current national guideline recommendations (AIOM-SIF Working Group, 2024), based on DPYD variants most strongly associated with reduced enzyme function, have improved the safety of fluoropyrimidine use, thanks to studies on variant-associated toxicity, most notably the meta-analysis by Meulendijks et al. (Pratt et al., 2024), which have been instrumental in preventing adverse events. Furthermore, the FUSAFE meta-analysis (Le Teuff et al., 2024) specifically demonstrated that the four recommended DPYD variants are the strongest predictors of grade 4–5 fluoropyrimidine toxicity — the most clinically critical events to prevent — further consolidating the rationale for pre-treatment genotyping. However, the sensitivity of our approach remains limited: the DPYD genotyping panel as for AIOM-SIF recommendations does not fully match the recent AMP consensus for clinical DPYD pharmacogenomic testing, which specifies a minimum Tier 1 set of 10 alleles and an extended Tier 2 list of 12 additional variants (Meulendijks et al., 2015). Future harmonization between national and international guidelines, potentially incorporating the AMP-recommended variant tiers, would enhance standardization and comprehensiveness of DPYD testing.

In this study we identified a large heterozygous deletion spanning from exon 5 to exon 20 of *DPYD* gene that was found to be associated with a strong decrease in the enzyme activity and likely to be related to the observed toxic event. In our case report the phenotypic effect of the observed genomic alteration was evaluated by assessing enzymatic activity in PBMCs which was found to be reduced by 49% as compared to two wild type healthy donors. This finding was further supported by the analysis of plasma U and DHU. Based on published data, a plasma uracil threshold of 16 ng/mL was used to discriminate between normal and deficient DPD activity, with DPD deficiency defined by U concentrations >16 ng/mL (Knikman et al., 2021; Contu et al., 2025). The DHU/U ratio was also considered to support the diagnosis of DPD deficiency, with values < 6 indicating impaired DPD activity, according to the literature (Contu et al., 2025; Capitain et al., 2020). Consistently, the patient samples showed increased plasma U concentrations and a reduced DHU/U ratio, supporting deficient DPD activity.

It has been previously documented that *DPYD* deletions account for approximately 7% of DPD deficiency cases (van Kuilenburg et al., 2009). Several cases have been described in which severe *fluoropyrimidine* toxicity was observed despite routine *DPYD* genotyping being negative and subsequently associated with rare intragenic deletions or other structural variants detectable only through extensive genomic analysis (van Kuilenburg et al., 2010; Wigle et al., 2023; Malekkou et al., 2024; Saarenheimo et al., 2021). In several cases, profound mucosal toxicity has been reported to precede systemic complications, including sepsis (García-González et al., 2018; de Haar-Holleman et al., 2024; Ly et al., 2020; Tong et al., 2018; Henricks et al., 2018b).

The likelihood of an association between the detected deletion and the occurrence of severe capecitabine-related toxicity is fully consistent with the patient’s clinical course, characterized by very early grade 4 diarrhoea, mucositis and metabolic alterations with consequent systemic complications shortly after exposure to *capecitabine* therapy. In a case series of nine patients, reduced DPD activity ranging from 9% to 53% of that observed in controls was reported due to novel and rare *DPYD* alterations. Severe toxicity ranging from grade 3 to lethal toxicity (grade 5), with onset after a mean of 11 days from the start of chemotherapy was observed in all patients (van Kuilenburg et al., 2017).

The residual DPD enzyme activity of approximately 50% in our patient is consistent with an intermediate metabolizer phenotype and indicates a reduced capacity to catabolize fluoropyrimidines. According to current pharmacogenetic recommendations, DPD activity in the range of 30%–70% of the mean control activity warrants an initial dose reduction, typically of 50%, rather than full-dose treatment. Accordingly, the enzymatic profile observed in this patient suggests an increased risk of fluoropyrimidine-related toxicity and does not support the assumption of normal drug tolerance. This case further underscores the clinical relevance of partial DPD deficiency in the prediction of treatment safety (Amstutz et al., 2018).

Furthermore, it should be highlighted that optimal capecitabine dose selection—given its complex, highly variable pharmacokinetics—cannot be based only on genetic markers (i.e., *DPYD* variants); instead, it requires integration of other parameters, such as renal and hepatic function, patient covariates (sex, age, body size), and absorption-related determinants, including strict prandial administration (within 30 min after a meal) (Ratain and O’Donnell, 2026; Knikman et al., 2024). In gastric cancer, prior partial/total gastrectomy is an additional key modifier, being associated with faster absorption and higher peak concentrations (reported C_max increases of ∼18% and ∼50%), plausibly increasing toxicity susceptibility and warranting heightened vigilance when individualizing therapy (Jacobs et al., 2019). Therefore, although the extensive deletion of the DPYD gene was probably the main factor underlying the severe capecitabine-related toxicity observed in this patient, other clinical factors, including the recent gastrectomy, the patient’s general clinical condition and inter-individual variability in the pharmacokinetics of capecitabine, may also have contributed to the severity of the adverse events.

The case described has important clinical implications given the adjuvant context in which chemotherapy has a curative purpose. In this context, treatment decision-making requires a careful balance between the potential oncological benefit and the acceptable risk. In fact, while definitive discontinuation of treatment could potentially compromise oncological benefit, the occurrence of early severe and potentially lethal toxicity represents an unacceptable risk in patients who could already be cured by surgery alone. This case therefore highlights the need to carefully stratify risk before initiating adjuvant fluoropyrimidine-based treatment based on comprehensive genomic and phenotypic data relating to the *DPYD* gene. Pre-treatment deletion detection would have indicated a 50% dose reduction per CPIC guidelines (Amstutz et al., 2018), mitigating severe toxicity risk. These findings support comprehensive genomic assessment, including copy number variant analysis and phenotypic confirmation, in patients with high planned fluoropyrimidine exposure or personal/family history of severe toxicity.

This case report provides further evidence that a sequencing-based (NGS) approach can strengthen pharmacogenetic testing. NGS enables comprehensive and less biased detection of pharmacogene variability, including rare or novel variants and structural gene aberrations. These alterations are inherently missed by targeted assays restricted to predefined panels. By overcoming limitations related to variant preselection, NGS offers broader allelic coverage. It also enables more consistent pharmacogenetic results across laboratories. This supports more equitable applicability across diverse ancestries, in which actionable allele spectra may differ. In addition, NGS allows future reanalysis of data as evidence bases and allele definitions evolve.

Despite these advantages, routine clinical implementation of NGS-based pharmacogenetics remains challenging. Relevant drawbacks include higher costs, infrastructure and expertise requirements, and workflow and turnaround-time constraints. A critical remaining issue is the lack of standardized variant interpretation and reporting (De Mattia et al., 2024). Furthermore, it should be considered that large structural variants in the DPYD gene are rare and that the benefits in terms of clinical outcomes of routinely performing comprehensive NGS testing have yet to be demonstrated. Several international initiatives are actively addressing these barriers with the aim of incorporating rare-variant evidence into pharmacogenomic clinical guidelines (Gong et al., 2025). Together with the increasing availability of NGS platforms and declining analytical costs, these efforts are expected to facilitate broader adoption of targeted pharmacogene sequencing, including *DPYD*, in routine clinical practice.

## 5 Conclusion

In conclusion, this case, based on robust molecular, biochemical and clinical evidence, provides compelling support for a causal relationship between the *DPYD* gene deletion, reduced DPD enzyme activity and consequent severe toxicity related to the use of fluoropyrimidines. The absence of a common *DPYD* variant in routine screening tests does not always equate to normal fluoropyrimidine metabolism. Structural alterations such as large multi-exon deletions are underestimated by these standard strategies, resulting in significant clinical consequences. Therefore, the use of comprehensive genomic techniques (extended genomic sequencing) combined with functional assays may help prevent potentially fatal toxicity, especially in selected settings such as adjuvant therapy.

## Data Availability

The original contributions presented in the study are included in the article/Supplementary Material, further inquiries can be directed to the corresponding author.

## References

[B1] AIOM-SIF Working Group (2024). Raccomandazioni per analisi farmacogenetiche. Available online at: https://www.aiom.it/wp-content/uploads/2024/11/2024_Racc-analisi-farmacogenetiche-update.pdf (Accessed April 27, 2026).

[B2] Amstutz U. Henricks L. M. Offer S. M. Barbarino J. Schellens J. H. M. Swen J. J. (2018). Clinical pharmacogenetics implementation consortium (CPIC) guideline for dihydropyrimidine dehydrogenase genotype and fluoropyrimidine dosing: 2017 update. Clin. Pharmacol. Ther. 103 (2), 210–216. 10.1002/cpt.911 29152729 PMC5760397

[B3] Bignucolo A. De Mattia E. Roncato R. Peruzzi E. Scarabel L. D'Andrea M. (2023). Ten-year experience with pharmacogenetic testing for *DPYD* in a national cancer center in Italy: lessons learned on the path to implementation. Front. Pharmacol. 14, 1199462. 10.3389/fphar.2023.1199462 37256229 PMC10225682

[B4] Capitain O. Seegers V. Metges J. P. Faroux R. Stampfli C. Ferec M. (2020). Comparison of 4 screening methods for detecting fluoropyrimidine toxicity risk: identification of the most effective, cost-efficient method to save lives. Dose Response 18 (3), 1559325820951367. 10.1177/1559325820951367 32973417 PMC7493257

[B5] Contu S. Launay M. Bouges Le Royer H. Simon L. Mignot A. Seutin E. (2025). Impact of renal and hepatic function on dihydropyrimidine dehydrogenase phenotype assessed by enzyme activity in peripheral blood mononuclear cells and uracilemia. Clin. Chem. Lab. Med. 64 (1), 166–175. 10.1515/cclm-2025-0949 40960875

[B6] de Haar-Holleman A. Cortoos P. J. Vlaeminck J. Van Landuyt P. Steurbaut S. Vaeyens F. (2024). Case report: a case of severe capecitabine toxicity due to confirmed in trans compound heterozygosity of a common and rare DPYD variant. Front. Pharmacol. 15, 1459565. 10.3389/fphar.2024.1459565 39376610 PMC11456491

[B7] De Mattia E. Silvestri M. Polesel J. Ecca F. Mezzalira S. Scarabel L. (2022). Rare genetic variant burden in DPYD predicts severe fluoropyrimidine-related toxicity risk. Biomed. Pharmacother. 154, 113644. 10.1016/j.biopha.2022.113644 36063648 PMC9463069

[B8] De Mattia E. Milan N. Assaraf Y. G. Toffoli G. Cecchin E. (2024). Clinical implementation of rare and novel *DPYD* variants for personalizing fluoropyrimidine treatment: challenges and opportunities. Int. J. Biol. Sci. 20 (10), 3742–3759. 10.7150/ijbs.97686 39113696 PMC11302886

[B9] De Mattia E. Park Y. Peruzzi E. Zhou Y. Roncato R. Polesel J. (2025). Integration of germline pharmacogenomic burden to predict fluoropyrimidine-related toxicity - a secondary analysis of the PREPARE trial. Oncogene 44 (45), 4352–4362. 10.1038/s41388-025-03587-7 40999005

[B10] Diasio R. B. Harris B. E. (1989). Clinical pharmacology of 5-fluorouracil. Clin. Pharmacokinet. 16 (4), 215–237. 10.2165/00003088-198916040-00002 2656050

[B11] Etienne-Grimaldi M. C. Pallet N. Boige V. Ciccolini J. Chouchana L. Barin-Le Guellec C. (2023). Current diagnostic and clinical issues of screening for dihydropyrimidine dehydrogenase deficiency. Eur. J. Cancer 181, 3–17. 10.1016/j.ejca.2022.11.028 36621118

[B12] European Medicines Agency (2020). EMA recommendations on DPYD testing prior to treatment with fluorouracil, capecitabine, tegafur and flucytosine. Available online at: https://www.ema.europa.eu/en/news/ema-recommendations-dpd-testing-prior-treatment-fluorouracil-capecitabine-tegafur-and-flucytosine (Accessed April 27, 2026).

[B13] García-González X. López-Tarruella S. García M. I. González-Haba E. Blanco C. Salvador-Martin S. (2018). Severe toxicity to capecitabine due to a new variant at a donor splicing site in the dihydropyrimidine dehydrogenase *(DPYD)* gene. Cancer Manag. Res. 10, 4517–4522. 10.2147/CMAR.S174470 30349384 PMC6190816

[B14] Gong L. Klein C. J. Caudle K. E. Moyer A. M. Scott S. A. Whirl-Carrillo M. (2025). Integrating pharmacogenomics into the broader construct of genomic medicine: efforts by the ClinGen pharmacogenomics working group (PGxWG). Clin. Chem. 71, 36–44. 10.1093/clinchem/hvae181 39749515 PMC12037359

[B15] Henricks L. M. Lunenburg C. A. T. C. de Man F. M. Meulendijks D. Frederix G. W. J. Kienhuis E. (2018a). DPYD genotype-guided dose individualisation of fluoropyrimidine therapy in patients with cancer: a prospective safety analysis. Lancet Oncol. 19 (11), 1459–1467. 10.1016/S1470-2045(18)30686-7 30348537

[B16] Henricks L. M. Siemerink E. J. M. Rosing H. Meijer J. Goorden S. M. I. Polstra A. M. (2018b). Capecitabine-based treatment of a patient with a novel DPYD genotype and complete dihydropyrimidine dehydrogenase deficiency. Int. J. Cancer 142 (2), 424–430. 10.1002/ijc.31065 28929491

[B17] Jacobs B. A. W. Deenen M. J. Joerger M. Rosing H. de Vries N. Meulendijks D. (2019). Pharmacokinetics of capecitabine and four metabolites in a heterogeneous population of cancer patients: a comprehensive analysis. CPT Pharmacometrics Syst. Pharmacol. 8 (12), 940–950. 10.1002/psp4.12474 31652031 PMC6930859

[B18] Knikman J. E. Gelderblom H. Beijnen J. H. Cats A. Guchelaar H. J. Henricks L. M. (2021). Individualized dosing of fluoropyrimidine-based chemotherapy to prevent severe fluoropyrimidine-related toxicity: what are the options? Clin. Pharmacol. Ther. 109 (3), 591–604. 10.1002/cpt.2069 33020924 PMC7983939

[B19] Knikman J. E. Lopez-Yurda M. Meulendijks D. Deenen M. J. Schellens J. H. M. Beijnen J. (2024). A nomogram to predict severe toxicity in DPYD wild-type patients treated with Capecitabine-Based anticancer regimens. Clin. Pharmacol. Ther. 115 (2), 269–277. 10.1002/cpt.3100 37957132

[B20] Knikman J. E. de With M. Heersche N. Lopez-Yurda M. Baars A. Creemers G. J. (2025). Dose-individualisation of fluoropyrimidines based on pre-treatment serum uracil levels: the Alpe2U study. Eur. J. Cancer 224, 115483. 10.1016/j.ejca.2025.115483 40466468

[B21] Larrue R. Fellah S. Hennart B. Sabaouni N. Boukrout N. Van der Hauwaert C. (2024). Integrating rare genetic variants into DPYD pharmacogenetic testing may help preventing fluoropyrimidine-induced toxicity. Pharmacogenomics J. 24 (1), 1. 10.1038/s41397-023-00322-x 38216550 PMC10786722

[B22] Le Teuff G. Cozic N. Boyer J. C. Boige V. Diasio R. B. Taieb J. (2024). Dihydropyrimidine dehydrogenase gene variants for predicting grade 4–5 fluoropyrimidine-induced toxicity: FUSAFE individual patient data meta-analysis. Br. J. Cancer 130, 808–818. 10.1038/s41416-023-02517-2 38225422 PMC10912560

[B23] Longley D. B. Harkin D. P. Johnston P. G. (2003). 5-fluorouracil: mechanisms of action and clinical strategies. Nat. Rev. Cancer 3 (5), 330–338. 10.1038/nrc1074 12724731

[B24] Ly R. C. Schmidt R. E. Kiel P. J. Pratt V. M. Schneider B. P. Radovich M. (2020). Severe capecitabine toxicity associated with a rare *DPYD* variant identified through whole-genome sequencing. JCO Precis. Oncol. 4, 00067. 10.1200/PO.20.00067 32923881 PMC7446391

[B25] Malekkou A. Tomazou M. Mavrikiou G. Dionysiou M. Georgiou T. Papaevripidou I. (2024). A novel large intragenic DPYD deletion causing dihydropyrimidine dehydrogenase deficiency: a case report. BMC Med. Genomics 17 (1), 78. 10.1186/s12920-024-01846-2 38528593 PMC10962175

[B26] Meulendijks D. Henricks L. M. Sonke G. S. Deenen M. J. Froehlich T. K. Amstutz U. (2015). Clinical relevance of *DPYD* variants c.1679T>G, c.1236G>A/HapB3, and c.1601G>A as predictors of severe fluoropyrimidine-associated toxicity: a systematic review and meta-analysis of individual patient data. Lancet Oncol. 16, 1639–1650. 10.1016/S1470-2045(15)00286-7 26603945

[B27] Pratt V. M. Cavallari L. H. Fulmer M. L. Gaedigk A. Hachad H. Ji Y. (2024). DPYD genotyping recommendations: a joint consensus recommendation of the association for molecular pathology, American college of medical genetics and genomics, clinical pharmacogenetics implementation consortium, college of American pathologists, Dutch pharmacogenetics working group of the royal Dutch pharmacists association, european society for pharmacogenomics and personalized therapy, pharmacogenomics knowledgebase, and pharmacogene variation consortium. J. Mol. Diagn 26, 851–863. 10.1016/j.jmoldx.2024.05.015 39032821 PMC12813241

[B28] Ratain M. J. O'Donnell P. H. (2026). Dear doctor: we still have no idea of what dose of capecitabine you should prescribe. J. Clin. Oncol. 44, 1186–1189. 10.1200/JCO-25-02896 41610386

[B29] Saarenheimo J. Wahid N. Eigeliene N. Ravi R. Salomons G. S. Ojeda M. F. (2021). Preemptive screening of DPYD as part of clinical practice: high prevalence of a novel exon 4 deletion in the Finnish population. Cancer Chemother. Pharmacol. 87 (5), 657–663. 10.1007/s00280-021-04236-y 33544210

[B30] Tong C. C. Lam C. W. Lam K. O. Lee V. H. F. Luk M. Y. (2018). A novel *DPYD* variant associated with severe toxicity of fluoropyrimidines: role of pre-emptive *DPYD* genotype screening. Front. Oncol. 8, 279. 10.3389/fonc.2018.00279 30087856 PMC6066555

[B31] van Kuilenburg A. B. Meinsma R. Zoetekouw L. Van Gennip A. H. (2002). Increased risk of grade IV neutropenia after administration of 5-fluorouracil due to a dihydropyrimidine dehydrogenase deficiency: high prevalence of the IVS14+1G>A mutation. Int. J. Cancer 101 (3), 253–258. 10.1002/ijc.10599 12209976

[B33] van Kuilenburg A. B. Meijer J. Mul A. N. Hennekam R. C. Hoovers J. M. de Die-Smulders C. E. (2009). Analysis of severely affected patients with dihydropyrimidine dehydrogenase deficiency reveals large intragenic rearrangements of DPYD and a *de novo* interstitial deletion del(1)(p13.3p21.3). Hum. Genet. 125 (5–6), 581–590. 10.1007/s00439-009-0653-6 19296131

[B34] van Kuilenburg A. B. Meijer J. Mul A. N. Meinsma R. Schmid V. Dobritzsch D. (2010). Intragenic deletions and a deep intronic mutation affecting pre-mRNA splicing in the dihydropyrimidine dehydrogenase gene as novel mechanisms causing 5-fluorouracil toxicity. Hum. Genet. 128 (5), 529–538. 10.1007/s00439-010-0879-3 20803296 PMC2955237

[B35] van Kuilenburg A. B. Meijer J. Maurer D. Dobritzsch D. Meinsma R. Los M. (2017). Severe fluoropyrimidine toxicity due to novel and rare DPYD missense mutations, deletion and genomic amplification affecting DPD activity and mRNA splicing. Biochim. Biophys. Acta Mol. Basis Dis. 1863 (3), 721–730. 10.1016/j.bbadis.2016.12.010 28024938

[B37] Wigle T. J. Medwid S. Ross C. Schwarz U. I. Kim R. B. (2023). *DPYD* exon 4 deletion associated with fluoropyrimidine toxicity and importance of copy number variation. Curr. Oncol. 30 (1), 663–672. 10.3390/curroncol30010051 36661700 PMC9857685

