# Supplementary material for: Identification of a large DPYD gene deletion undetected by standard genotyping in a patient with severe capecitabine-related toxicity: a case report
Source: Front Pharmacol. 2026 Aug 4;17:1866621. doi: 10.3389/fphar.2026.1866621 (PMC13481457; doi:10.3389/fphar.2026.1866621)
Supplement: Supplementary file 1 [file Supplementaryfile1.docx]

Supplementary Material

**Methods**

**DNA extraction and genotyping (RT-PCR)**

Molecular analysis of the *DPYD* gene was performed on ethylenediaminetetraacetic acid (EDTA) whole blood processed within 30 minutes or within 5 days from storage at 4°C. DNA extraction from PBMC was performed using a Qiagen’s QIAamp DNA Blood Kit (61104, Qiagen) according to the manufacturer’s protocol. DNA was subsequently quantified using the Invitrogen Qubit 4 Fluorometer (Q33238, Thermofisher Scientific).

DNA samples were genotyped and analyzed for 5 *DPYD* gene variants using EasyPGX® ready *DPYD* kit (RT026, Diatech Pharmacogenetics, Italy) and “EasyPGX *DPYD* Analysis Software” version 4.0.1 (both CE-IVD marked) according to the manufacturer’s protocol. Through allelic discrimination the test allows the detection of the 4 main variants validated as clinical markers associated with drug toxicity according to the EMA. Agenzia Italiana del Farmaco (AIFA) plus *DPYD* c.2194GA exclusively recommended by AIOM-SIF recommendations in the post-toxicity setting [14].

**NGS**

***SureSelect target enrichment library prep system with Custom capture library RNA baits design***

The SureSelect custom capture library baits were synthesized and designed by Agilent Technologies (Agilent, Santa Clara, CA, USA) by using SureDesign (Agilent, Santa Clara, CA, USA). Probes were designed using the H. sapiens reference genome Build Version UCSC hg38, GRCh38, December 2013. RNA probes were placed to target genomic regions that include 94 genes CDS, 2067 SNPs as well as intronic and UTR regions for specific genes, such as *CYP2D6*, generating a final panel size of circa 595 kbp. RefSeq, Ensembl, CCDS, Gencode, VEGA, SNP, CytoBand database have been used for the panel design, considering a region extension of 25 bp for the exon-intron junction.

***Library preparation, target DNA enrichment, and NGS sequencing***

Library preparation, hybridization and enrichment of DNA extracted from blood samples was performed using the SureSelect XT HS2 target enrichment system for Illumina paired-end sequencing libraries (Agilent, Santa Clara, CA, USA) according to manufacturer’s instructions (SSELXT-HS2). Next, each library was hybridized to the H. sapiens-specific RNA-baits based custom panel and final libraries were assessed using an Agilent TapeStation using high sensitivity D 1000 ScreenTape and then pooled for Illumina sequencing. Sequencing of SureSelect enriched libraries was performed using Illumina MiSeq platform using v2 300-cycle cartridges (2 × 150 bp) following manufacturer’s guidelines (Illumina, Inc., San Diego, CA, USA). Sequencing data were processed using NGS secondary analysis Alissa Reporter (Agilent, Santa Clara, CA, USA), which included modules for alignment and variant calling. NGS tertiary analysis was performed on Alissa Interpret software (Agilent, Santa Clara, CA, USA) for annotation.

***Bioinformatics pipeline for CNV detection***.

CNV analysis was initially performed using Alissa Reporter (Agilent Technologies), a cloud-based secondary analysis platform for NGS data that enables detection of SNVs, indels, and CNVs from FASTQ files. CNV calling in Alissa Reporter is based on a read-depth approach at exon-level resolution, leveraging coverage information derived from aligned BAM files. More specifically, the algorithm performs CNV detection by:

• Using post-processed BAM files (including positional deduplication and filtering of reads with low mapping quality)

• Calculating relative coverage differences between the target sample and a reference set

• Estimating log2 ratios for each targeted interval (e.g., exon)

• Identifying CNVs as deviations from the expected diploid coverage profile.

Importantly, Alissa Reporter applies a co-analysis strategy, in which the reference signal is dynamically derived from a group of unrelated samples sequenced within the same run. This approach eliminates the need for a predefined reference sample while enabling robust normalization. For optimal performance, at least three samples (preferably eight or more) are analyzed together to construct a reliable reference baseline.

This strategy allows accurate detection of CNVs across a wide range of sizes, including single-exon events, by comparing normalized read depth of each interval against the expected coverage derived from the cohort. Given the discontinuation of Alissa Reporter, the CNV identified in this study was subsequently re-evaluated using the SeqOne platform (SeqOne Genomics, Montpellier, France) to ensure robustness and reproducibility. In SeqOne, CNV detection was performed using the CNV Capture v2.6 application, which also relies on a read-depth–based method applied to targeted sequencing data. The underlying algorithm (app_cnv) evaluates coverage changes across captured regions to infer copy number alterations. The CNV detected using Alissa Reporter was fully concordant with the result obtained using SeqOne, thereby supporting the reliability of the finding across two independent bioinformatics analysis.

**SNP Array**

SNP array was performed using the Infinium 850K BeadChip Array platform (Illumina, San Diego, CA). An oligonucleotide-based microarray with an overall average effective resolution of 18 kb to detect CNVs. A total of 200 ng DNA from the patient was processed, labeled and hybridized according to the manufacturer’s instruction (http://www.illumina.com). Array data files were imported into BlueFuse Multi version 4.5 (Illumina) and analyzed using the default BlueFuse algorithm and settings (which requires a minimum of 10 clones for CNV and a minimum of 200 clones or 3 MB for LOH) by the evaluation of signal intensity (log R ratio) and allelic composition (B Allele Frequency).

**DPD activity in PBMCs**

Samples were collected at two time points using EDTA-anticoagulated blood, as previuosly described [36]: the first approximately six months after capecitabine therapy had been discontinued, and the second approximately nine months after treatment discontinuation. PBMCs were isolated as previously described by van Kuilenburg et al. [36, 37]. The activity of DPD was determined in an assay mixture containing 0.1 M HEPES (pH 7.3), 2.5 mM MgCl2 , 250 µM NADPH, 1 mM dithiothreitol and 25 µM thymine. After one hour of incubation, the reaction was terminated by the addition of formic acid and the amount of dihydrothymine was determined using tandem-mass spectrometry.

**Uracil and DHU determination in plasma**

Samples were collected at two time points using EDTA-anticoagulated blood, as previuosly described [36]: the first approximately six months after capecitabine therapy had been discontinued, and the second approximately nine months after treatment discontinuation. Uracil and DHU concentrations in plasma were measured using the DOSIURA kit (Shimadzu Corporation, Tokyo, Japan) following the manufacturer’s instructions and using the reagents provided in the kit. Type I ultrapure water was obtained from a Milli-Q Advantage A10 system (Millipore, Billerica, MA, USA). Chromatographic separation was performed on a Nexera LC-40 X3 HPLC system comprising a SIL-40C X3 CL autosampler, three LC-40D X3 CL pumping modules, two DGU-405 CL degassers, a CBM-40 CL system controller, and a CTO-40C CL column oven. The HPLC system was coupled to a CITRINE 6500 QTrap triple quadrupole mass spectrometer (SCIEX, Massachusetts, USA) equipped with a Turbo Ion Spray source operating in positive ion mode. Each analyte and internal standard was quantified by multiple reaction monitoring using two transitions per compound: one for quantification (quantifier) and one for confirmation (qualifier). Data acquisition and processing were performed with Skyline software (version 25.1.0.237. 64-bit). Sample preparation was carried out according to the manufacturer’s protocol. Briefly: 100 µL of plasma were thawed at room temperature and internal standard solution was added. Proteins were precipitated with DOSIURA Extraction buffer and the mixture was vortexed for 1 min and centrifuged at 15.000 × g for 10 min. A 30 µL aliquot of the supernatant was diluted with 270 µL of water, vortexed and injected into the LC-MS system.

| gene_name | chr | start | end | exon_intron_number | status | type | copy_ratio |
| --- | --- | --- | --- | --- | --- | --- | --- |
| DPYD | 1 | 97921034 | 97922034 | 0 | normal | upstream | 0,91 |
| DPYD | 1 | 97920902 | 97921053 | 1 | normal | exon-UTR | 0,98 |
| DPYD | 1 | 97920863 | 97920941 | 1 | normal | exon-CDS | 0,98 |
| DPYD | 1 | 97883374 | 97920882 | 1.5 | normal | intron | 0,83 |
| DPYD | 1 | 97883243 | 97883393 | 2 | normal | exon-CDS | 0,80 |
| DPYD | 1 | 97828196 | 97883262 | 2.5 | normal | intron | 0,76 |
| DPYD | 1 | 97828093 | 97828215 | 3 | normal | exon-CDS | 0,85 |
| DPYD | 1 | 97740479 | 97828111 | 3.5 | normal | intron | 0,88 |
| DPYD | 1 | 97740371 | 97740498 | 4 | normal | exon-CDS | 0,95 |
| DPYD | 1 | 97721671 | 97721939 | 4.5 | normal | intron | 0,77 |
| DPYD | 1 | 97721489 | 97721690 | 5 | deletion | exon-CDS | 0,60 |
| DPYD | 1 | 97719947 | 97721507 | 5.5 | deletion | intron | 0,50 |
| DPYD | 1 | 97699330 | 97699566 | 6 | deletion | exon-CDS | 0,50 |
| DPYD | 1 | 97691798 | 97699348 | 6.5 | deletion | intron | 0,42 |
| DPYD | 1 | 97691696 | 97691817 | 7 | deletion | exon-CDS | 0,49 |
| DPYD | 1 | 97679182 | 97679414 | 7.5 | failed | intron | 0,00 |
| DPYD | 1 | 97679074 | 97679201 | 8 | failed | exon-CDS | 0,00 |
| DPYD | 1 | 97678866 | 97679092 | 8.5 | deletion | intron | 0,32 |
| DPYD | 1 | 97595038 | 97595185 | 9 | deletion | exon-CDS | 0,59 |
| DPYD | 1 | 97593387 | 97595057 | 9.5 | deletion | intron | 0,44 |
| DPYD | 1 | 97593197 | 97593406 | 10 | deletion | exon-CDS | 0,48 |
| DPYD | 1 | 97573970 | 97580016 | 10.5 | deletion | intron | 0,43 |
| DPYD | 1 | 97573739 | 97573989 | 11 | deletion | exon-CDS | 0,47 |
| DPYD | 1 | 97549744 | 97573758 | 11.5 | deletion | intron | 0,45 |
| DPYD | 1 | 97549539 | 97549763 | 12 | deletion | exon-CDS | 0,44 |
| DPYD | 1 | 97515941 | 97549557 | 12.5 | deletion | intron | 0,42 |
| DPYD | 1 | 97515705 | 97515960 | 13 | deletion | exon-CDS | 0,50 |
| DPYD | 1 | 97507523 | 97515723 | 13.5 | failed | intron | 0,00 |
| DPYD | 1 | 97450038 | 97450242 | 14 | deletion | exon-CDS | 0,50 |
| DPYD | 1 | 97382461 | 97402357 | 14.5 | deletion | intron | 0,48 |
| DPYD | 1 | 97382372 | 97382480 | 15 | deletion | exon-CDS | 0,46 |
| DPYD | 1 | 97373644 | 97382390 | 15.5 | failed | intron | 0,00 |
| DPYD | 1 | 97373540 | 97373663 | 16 | deletion | exon-CDS | 0,49 |
| DPYD | 1 | 97306297 | 97373559 | 16.5 | deletion | intron | 0,52 |
| DPYD | 1 | 97306156 | 97306316 | 17 | deletion | exon-CDS | 0,54 |
| DPYD | 1 | 97305378 | 97306174 | 17.5 | deletion | intron | 0,51 |
| DPYD | 1 | 97305238 | 97305397 | 18 | deletion | exon-CDS | 0,49 |
| DPYD | 1 | 97234994 | 97305256 | 18.5 | deletion | intron | 0,51 |
| DPYD | 1 | 97234831 | 97235013 | 19 | deletion | exon-CDS | 0,50 |
| DPYD | 1 | 97193048 | 97193267 | 20 | deletion | exon-CDS | 0,51 |
| DPYD | 1 | 97137332 | 97137566 | 20.5 | normal | intron | 0,74 |
| DPYD | 1 | 97098468 | 97098651 | 21 | normal | exon-CDS | 0,85 |
| DPYD | 1 | 97082309 | 97082489 | 22 | normal | exon-CDS | 0,84 |
| DPYD | 1 | 97082146 | 97082328 | 22.5 | normal | intron | 0,89 |
| DPYD | 1 | 97078955 | 97079165 | 23 | normal | exon-CDS | 0,94 |
| DPYD | 1 | 97077892 | 97078994 | 23 | normal | exon-UTR | 0,94 |

*Supplementary Table S1*. SeqOne analysis of copy number profile for DPYD gene: a marked decrease in copy number is observed across exons 5 to 20 (highlighted), consistent with a heterozygous deletion, while flanking exons display values near the diploid state. Exon-level copy ratios ~0.4–0.6 across exons 5–20 and diploid values in flanking regions are reported above.

**References**

[36] van Kuilenburg, A. B., Van Lenthe, H., Tromp, A., Veltman, P. C., and Van Gennip, A. H. (2000). Pitfalls in the diagnosis of patients with a partial dihydropyrimidine dehydrogenase deficiency. *Clin. Chem.* 46 (1), 9–17.

[37] van Kuilenburg, A. B. P., Pleunis-van Empel, M. C. H., Brouwer, R. B., Sijben, A. E. J., Knapen, D. G., Oude Munnink, T. H., et al. (2024). Lethal Capecitabine Toxicity in Patients With Complete Dihydropyrimidine Dehydrogenase Deficiency Due to Ultra-Rare *DPYD* Variants. *JCO Precis. Oncol.* 8, e2300599. doi:10.1200/PO.23.00599
